# Photochemistry of tetra- through hexa-brominated dioxins/furans, hydroxylated and native BDEs in different media

**DOI:** 10.1007/s11356-015-5065-7

**Published:** 2015-08-11

**Authors:** Marek Roszko, Krystyna Szymczyk, Renata Jędrzejczak

**Affiliations:** Department of Food Analysis, Institute of Agricultural and Food Biotechnology, Rakowiecka 36, 02-532 Warsaw, Poland

**Keywords:** Brominated dioxins, PBDE, OH-BDE, Degradation, UV

## Abstract

The aim of this study was to investigate (i) the behavior of native PBDEs during UV irradiation in different media, (ii) the possibility of their transformation into hydroxylated PBDEs in aqueous media, and (iii) the photochemistry/levels of brominated dioxins/furans formed from hydroxylated PBDEs. Debromination leading to the formation of a wide range of low-brominated congeners was the main path of photocatalyzed transformations of PBDEs. In organic solvents other than toluene, BDEs degraded in line with the pseudo first order kinetics (10–20 min half-life, depending on congener type and reaction medium). Irradiated BDE 209 congener behaved quite differently than lower-brominated BDEs: detectable amounts of various bromo-benzenes were found. That suggests that UV irradiation of BDE 209 leads to cleavage of the ether bound between the congener’s aromatic rings. Formation of bromophenyl bromo-methyl-biphenyl ethers or benzyl-bromophenoxybenzenes was observed in irradiated PBDE toluene-based solutions. The total concentration of OH-BDEs found in the reaction medium did not exceed 0.2 % of the initial precursor mass. Moreover, lower-brominated congeners detected in the reaction medium indicate subsequent debromination of OH-BDEs or hydro-debromination of the degraded congeners. Brominated dioxins and low levels of furans were observed in samples containing OH-BDEs. The total mass of dioxins did not exceed 3.5 % of the initial precursor mass.

## Introduction

Polybrominated diphenyl ethers (PBDE) are flame retardant agents with numerous industrial applications. Even if in majority of developed countries they have been banned for some time, elevated levels of those compounds are still observed in environmental/biota/human tissue samples (Chen et al. 2012); some authors report still an upward trend. (Bio) accumulative behavior of those compounds and suspected endocrine disrupting potency raises some serious concerns about their presence in the environment (Canton et al. 2008; Dingemans et al. 2008). In addition, it was concluded that PBDEs released into the environment could transform into a wide spectrum of analogue chemicals.

Low photo stability of PBDEs has been reported in a number of studies (Hernik et al. 2007). Debromination process caused by UV irradiation (leading to wide range of lower-brominated congeners) is described in details. Brominated dioxins and furans (PBDD/Fs) were found in various plastic materials containing PBDEs (Ortuno et al. 2014). PBDD/Fs are also known to be produced when materials containing PBDEs are incinerated (Ortuno et al. 2015). Some reports suggest that native compounds may transform into hydroxylated PBDEs (Rayne et al. 2006). OH-substituted congeners are also known biotransformation products of native PBDEs. Such derivatives might be also formed during (photo) oxidation reactions running in native compounds (Hamers et al. 2008; Ueno et al. 2008).

Some recent studies have indicated that polybrominated dibenzo-p-dioxins and dibenzofurans might be formed from hydroxylated PBDE congeners in UV-catalyzed ring closure reaction (Haglund et al. 2007; Steen et al. 2009; Arnoldsson et al. 2012; Erickson et al. 2012). In addition, elevated levels of brominated dioxins have been reported in some marine organisms (Marsh et al. 2004; Malmvarn et al. 2008; Houde et al. 2009; Lofstrand et al. 2010). However, sparse available reports do not suggest that brominated dioxin levels/profiles in biota/sediments seem to reflect elevated levels of dioxins and hydroxylated PBDE (most probably of natural origin) in low trophy marine organisms. Those divergent observations have not helped to identify pathways of those compounds in the environment. Recent reports on brominated dioxin/furans identified in marine atmosphere and on similarity of their profiles to profiles observed in biota suggest rather exposure from some anthropogenic sources (Chao et al. 2014).

Despite quite a few reports on photochemical behavior of PBDEs are available, quantitative data on possibility of formation of hydroxylated derivatives from native compounds at their actual concentrations in the environment (low ng ml^−1^ range) are still missing. As noticed by Rayne et al. (2006), data obtained at high levels of PBDEs do not obviously reflect processes occurring in the environment. Also, reports on photochemical behavior of brominated dioxins and furans are missing.

The aim of this study was to investigate behavior of native PBDEs during UV irradiation in various media. The possibility that native congeners in aqueous media may transform into hydroxylated PBDEs was assessed. Finally, levels of brominated dioxins/furans formed from hydroxylated PBDEs and their photochemistry have been evaluated.

## Materials and methods

### Chemicals/reagents

Native PBDE standards (IUPAC 1, 2, 3, 7, 8, 10, 11, 12, 13, 15, 17, 25, 28, 30, 32, 33, 35, 37, 47, 49, 66, 71, 75, 77, 85, 99, 100, 110, 116, 118, 119, 126, 138, 153, 154, 155, 166, 181, 183, 190, 209), hydroxylated BDEs (3-OH-BDE 28, 3-OH-BDE 47, 6-OH-BDE 47, 6-OH-BDE 99, 5-OH-BDE 99, 3-OH-BDE 154, 6-OH-BDE 137, 6-OH-BDE 180) were supplied by AccuStandard (New Haven, CT, USA). Native PBDD/Fs (2,3,7,8-TBDD, 1,2,3,7,8-PeBDD, 1,2,3,4,7,8-HxBDD, 1,2,3,6,7,8-HxBDD, 1,2,3,7,8,9-HxBDD, 1,2,3,4,6,7,8-HpBDD, OBDD, 2,3,7,8-TBDF, 1,2,3,7,8-PeBDF, 1,2,3,4,7,8-HxBDF, 1,2,3,6,7,8-HxBDF, 1,2,3,7,8,9-HxBDF, 1,2,3,4,6,7,8-HpBDF) and ^13^C_12_ PCB 181 standards were obtained from Cambridge Isotope Laboratories. Water was purified using HydroLab (Wislina, Poland) water treatment system.

### Irradiation experiments

Studied BDE congeners (28, 47, 99, 100, 153, 154, 183, and 209) were dissolved in n-hexane to a final concentration of 50 μg ml^−1^. OH-BDE (3-OH-BDE 28, 3-OH-BDE 47, 6-OH-BDE 47, 6-OH-BDE 99, 5-OH-BDE 99, 3-OH-BDE 154, 6-OH-BDE 137, 6-OH-BDE 180) standard solutions were prepared at the same concentration level using acetonitrile as solvent.

Irradiation experiments were performed using a 4-W mercury UV lamp operated at 254 nm wavelength; 500 ng (in most cases) portions of the investigated substance were dissolved in organic solvents or aqueous solutions, placed in reaction vials made of borosilicate glass and irradiated at approx. 16 mW cm^−2^. Directly after processing, samples were transferred into amber chromatographic vials. In case of aqueous solutions, extraction into n-hexane was performed. Alkaline solutions were acidified down to pH 2 with acetic acid before extraction.

Chromatographic analysis of the samples proved that no additional cleanup of the reaction mixtures was necessary. Native BDEs, their degradation products, and PBDD/Fs were determined using GC/IT-MS. OH-BDEs were determined using LC/TOF-MS.

### Chemical analysis

Samples were analyzed using a Thermo-Finnigan Trace GC Ultra gas chromatograph (Austin, TX, USA) connected via a heated transfer line with a Polaris Q low-resolution ion-trap mass spectrometer (Austin, TX, USA) equipped with a Programmable Temperature Vaporizer (PTV)-based injector, TriPlus Autosampler (Austin, TX, USA). Chromatographic separations were performed on a 20 m × 0.18 mm × 0.1 μm Rtx-5 MS 5 %-Phenyl-fused-silica capillary column (Restek, Bellefonte, PA, USA) connected via a Vu2 Union connector (Restek, Bellefonte, PA, USA) to a 5 m × 0.53 mm guard column/retention gap (Restek). The mass spectrometer transfer line and ion source were kept at 320 and 300 °C, respectively. Mass calibration was tuned against perfluorotributylamine (FC-43) in electron-impact positive ionization mode according to the manufacturer’s recommendations. Detailed mass spectrometer operating parameters were reported previously (Roszko et al. 2013).

Acquity H-Class ultra-high performance liquid chromatograph coupled to a LCQ Premiere XE time of flight high resolution mass spectrometer Waters (Milford, MA, USA) was used for the purpose of this study. Chromatographic separations were performed on a non-porous (core-shell) PFP column 10 cm × 2.1 mm × 1.7 μm (Phenomenex, Bellefonte, PA, USA). Mobile phase composed of water and methanol both containing 4 mM of ammonium formate was used. Determinations were performed using an ESI source operated in negative polarity. Ion source and the de-solvation temperatures were set at 150 and 350 °C, respectively. Nebulizing gas (nitrogen) flow rate was set at 750 l min^−1^ while the cone gas flow rate was set at 20 l min^−1^. Capillary voltage was 3000 V and the ion optics was operated in the V mode.

### QA/QC

^13^C_12_ PCB 181 was used as the syringe standard. Due to commonly reported problems with laboratory background in the vicinity of the BDE 209 peak, two blank samples were analyzed with every batch of samples. Some laboratory background was observed but it was small with respect to the observed BDE levels. The results were corrected against mean value of the two blanks.

Commercially available PBDE, PBDD/F, and OH-BDE standard mixtures were used to assess method statistical parameters. Calibration curves were plotted as response ratio (height of the analyte peak to height of the syringe standard peak) versus analyte concentration. Each curve was forced to pass through the origin (0,0 point).

Xcalibur version 1.2 and MassLynx 4.1 software programs were used to acquire and analyze data. Individual results are given as a mean value of three parallel determinations (±1 SD, if given). Due to limited number of authentic standards, some chromatographic peaks were identified based on some literature data. Quantifications were based on the response factor calculated for the chromatographic peak with the nearest retention time. Those compounds have been indicated as tentative structures. The results were statistically analyzed using the Statistica 9.0 software package.

## Results and discussion

### UV irradiation-induced debromination of PBDEs

Available literature data clearly indicate that PBDEs exposed to UV irradiation easily degrade, mainly by way of debromination. However, only a limited number of produced congeners was quantified in majority of published papers (Hua et al. 2003; Raloff 2003; Eriksson et al. 2004). Moreover, majority of those studies have been performed using relatively high PBDE initial concentrations, not corresponding to actual environmental/biota levels. As noticed by Rayne et al. (2006), the reaction medium may significantly affect range of the formed products and the overall reaction rate in view of the reductive debromination nature of the running reactions. In this respect, results obtained on samples irradiated in n-hexane may not reflect processes occurring in aqueous environments or in the atmosphere.

Results of this study prove that debromination leading to formation of a wide range of low-brominated congeners was the main path of photocatalyzed transformations of PBDEs. UV irradiation decreased also rather quickly the overall mass of PBDEs in the reaction mixture, which resulted not only from loss of bromine atoms.

**Fig. 1:**
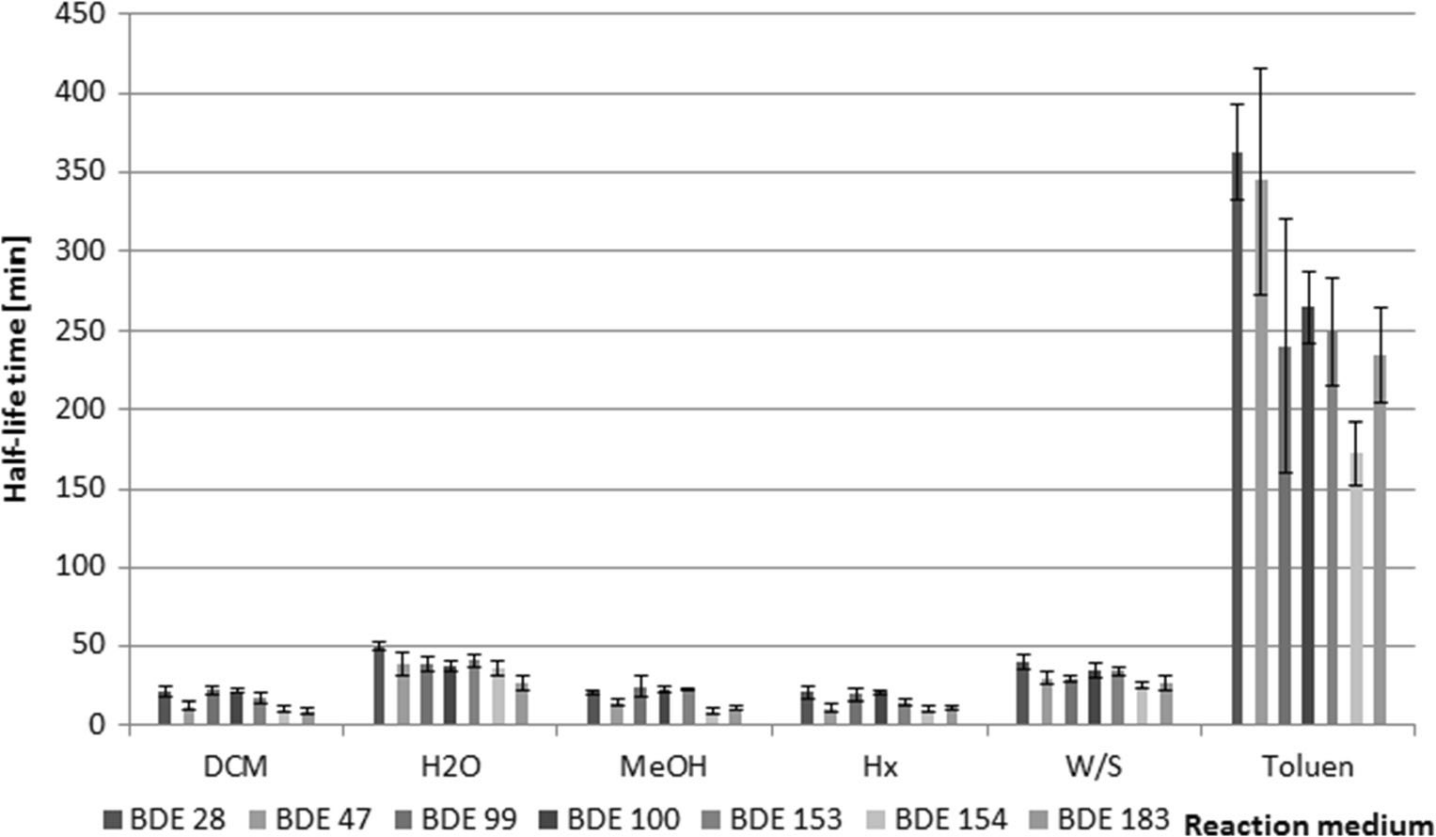
Mean determined half-life time of the selected seven studied congeners irradiated in six various media

**Fig. 2:**
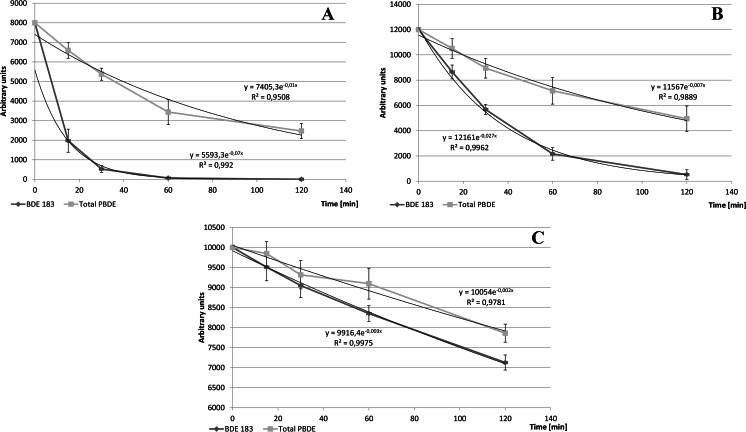
Drop of BDE183 and total PBDE concentration in dichloromethane (**a**), water (**b**), and toluene (**c**) with time of UV irradiation

In organic solvents other than toluene, BDEs degraded in line with the pseudo first order kinetics. Half-life was typically 10–20 min, depending on congener type and reaction medium, see Fig. 1. After that time, 63–77 % of the initial precursor mass remained in the reaction medium. Drop of BDE183 and total BDEs concentration in dichloromethane, water, and toluene with time of UV irradiation is shown in Fig. 2a–c, respectively.

Rayne et al. (2006) observed an 85 % drop in initial concentration of BDE 153 in 1.3 × 10^−9^ M acetonitrile solution after 5 min of irradiation with a 6-W UV lamp. Penta-brominated BDEs were the dominant degradation products at approx. 14 % share of the precursor mass. Their calculated average share in the reaction mixture was in the 39–45 % range (depending on the irradiation time). However, after irradiation time corresponding to 85 % degradation of the precursor, penta-BDEs represented only 5 % of the precursor mass. The observed profiles were generally in line with the literature.

**Fig. 3:**
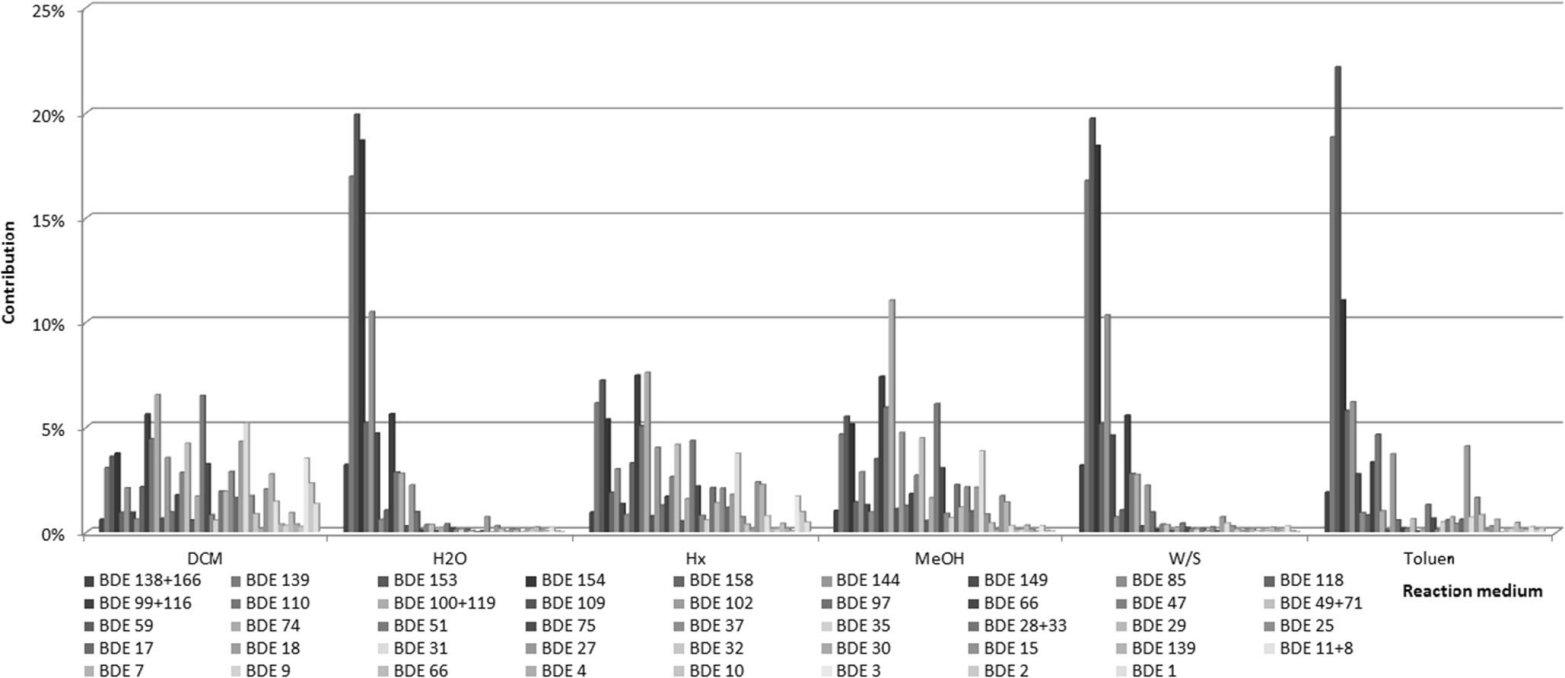
Observed mean BDE profiles after UV irradiation of BDE 183 in six various media

Rapid drop in BDE concentration was observed in organic solvents other than toluene (compare Fig 2a and b with c). The reaction medium significantly affected also profiles of the formed BDEs (Fig. 3).

PBDEs in the used concentration range are soluble in the used solvents. Izadifard et al. (2008) reported that photo-debromination induced in alcohol produced shifted congener profiles. We did not observe any a similar effect in methanol used as the solvent. Significant differences in profiles and the reaction speed were observed when samples were irradiated without any solvent, or when they were mixed with water. In the latter case, the reaction speed was even more significantly reduced. PBDEs solubility in water is extremely low, and the debromination reaction runs only on surfaces of solid particles rather than in the entire volume as in the case of an organic solvent. The debrominated congeners formed from precursors were subsequently further degraded to simple compounds like diphenyl ether or bromophenols, so the degradation product profile was relatively narrow. Additionally, a relatively small bias in concentration of the precursor BDE and the total BDE mass in the reaction mixtures was also observed. Reaction half-life times observed in water were even higher than those observed in systems without any solvent, most probably because of reflection/dispersion/absorption of UV light in water. Relatively higher stability of PBDEs in water was also reported by Sanchez-Prado et al. (2006). Contrary to that, Rayne et al. (2006) suggested that water promoted degradation of PBDEs: they suggested that some nucleophilic reaction occurring during irradiation of BDE 153 in water led to formation of hydroxylated BDEs and that way significantly affected the observed mass balance. However, no such behavior was observed in this study.

In line with Eriksson et al. (2004) and Palm et al. (2004), we found that higher brominated congeners (that exhibit higher absorption rates for UV radiation than lower-brominated ones) were more susceptible to photo-debromination. According to Bezares-Cruz et al. (2004), ortho-substituted congeners show the lowest photolytic stability.

Irradiation experiments performed with BDE 209 gave significantly different results than those performed with lower-brominated BDEs. Half-life times observed for BDE 209 were in the 4–86-min range, depending on the solvent (the highest value for the toluene-based solution). Detectable quantities of nona- and octa-brominated BDEs and bromobenzene were observed in reaction mixtures. Bromobenzene suggests UV-induced cleavage of ether bound between BDE 209 aromatic rings. This is in agreement with Watanabe and Tutsukawa (1987). Shih and Wang (2009) reported significant quantities of octa- and nona-substituted BDEs when BDE 209 was irradiated; however, they used significantly higher precursor concentrations.

### Irradiation of PBDEs in toluene

**Fig. 4:**
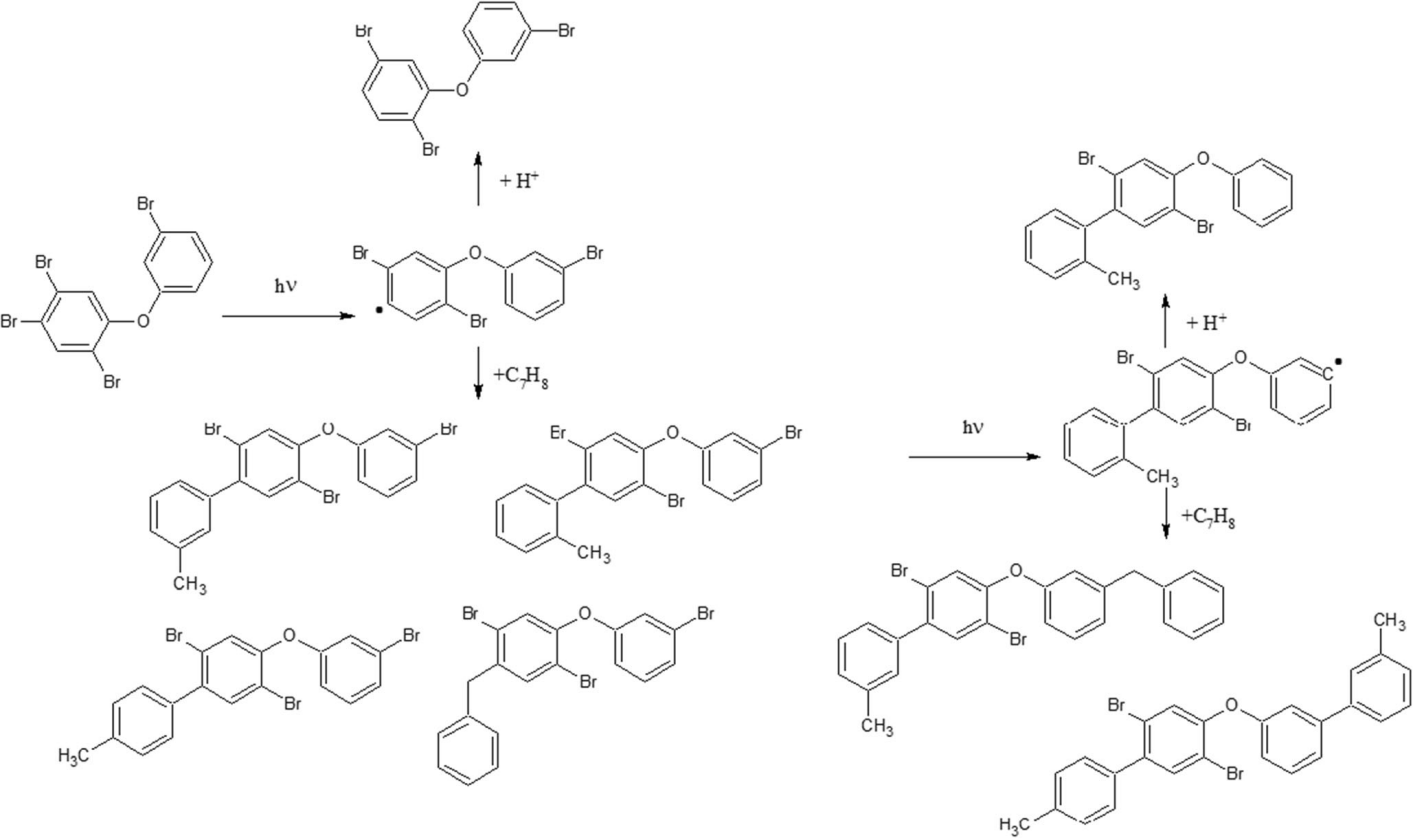
A possible scheme of BDEs reaction with toluene under UV irradiation

**Fig. 5:**
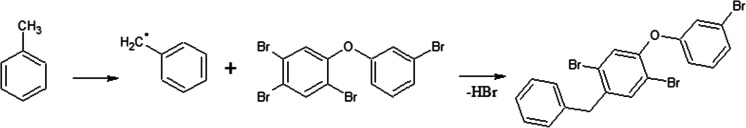
Alternative scheme of BDEs reaction with toluene under UV irradiation (the reaction initiated by solvent radical)

Results of this study indicate that UV-induced reactions run quite differently when PBDEs are dissolved in toluene then in other organic solvents. Toluene’s higher absorption rate for UV radiation might explain longer half-life times for PBDE degradation reactions running in toluene. However, chromatographic analysis revealed significant quantities of compounds formed in reaction of toluene with PBDEs. Those were identified as “adducts” of the toluene molecule to the polybrominated diphenyl ether, namely bromophenyl bromo-methyl-biphenyl ethers or benzyl-bromophenoxybenzenes. Most probably they were formed by way of the radical substitution reaction. A possible reaction scheme is shown in Fig. 4: PBDEs bromine atoms cleave due to high reactivity under UV irradiation, simultaneously formed aryl radicals (Suh et al. 2009) attack toluene molecules. However, Suh et al. (2009) observed radicals in UV-irradiated pure toluene, which may indicate that this reaction is initiated by solvent molecule (Fig. 5).

**Fig. 6:**
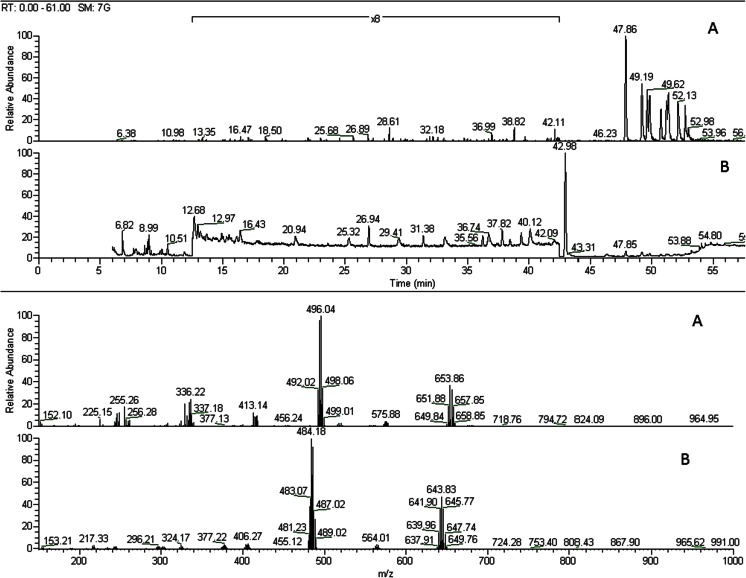
Chromatograms (*top*) and the corresponding mass spectra (*bottom*) of BDE/toluene reaction products under UV irradiation. **a** BDE 153. **b** BDE 47

Regardless of the actual reaction scheme, spectrum of products formed in any radical substitution reaction should be wide. The results of this study confirm this: a wide spectrum of precursor molecules substituted with toluene was observed in the reaction mixtures. BDEs substituted with more than one toluene molecules were even found. Typical chromatogram and mass spectra of products of BDE 153/toluene reactions are shown in Fig. 6. Similar products were observed for all studied BDE congeners except BDE 209. In the latter case, no such products were identified in the reaction mixtures. This seems to confirm different scheme of BDE 209 degradation process.

**Fig. 7:**
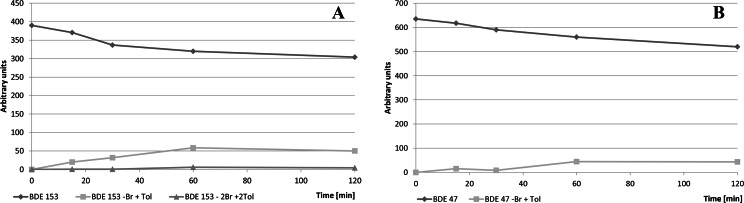
Change of BDE/toluene reaction product concentrations with time of UV irradiation. **a** BDE 153. **b** BDE 47 \

Time evolution of concentrations of BDE 153/47 and their reaction products with toluene during UV irradiation are shown in Fig. 7a, b, respectively. Concentrations of reaction products do not clearly rise, which suggests that these compounds also degrade. Generally, higher concentrations of reaction products were observed for higher brominated congeners, which (most probably) just reflected their higher photoreactivity.

### Hydroxylated PBDEs and brominated dioxins/furans

**Fig. 8:**
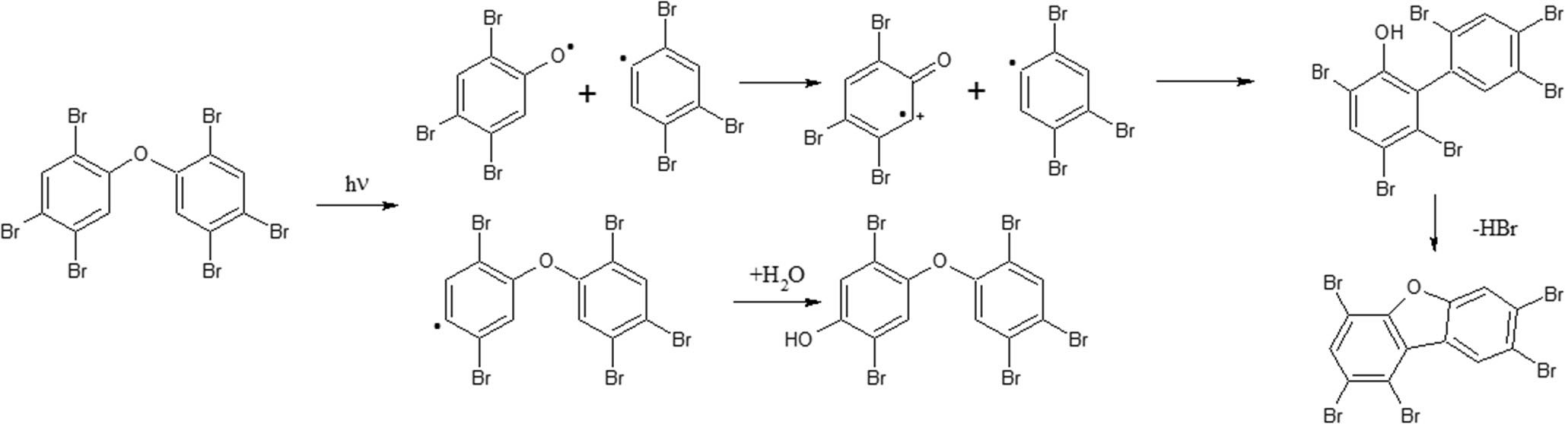
Proposed scheme of OH-BDEs/OH-BBs formation from native BDEs under UV irradiation

Freeman and Srinivas (1983) and Rayne et al. (2006) suggested that PBDEs irradiated in aqueous media could degrade/transform into hydroxylated molecules. OH-BDEs might be formed from other degradation products like bromophenols in a similar manner as reported for PCBs (Routti et al. 2008; Routti et al. 2009) (see Fig. 8).

**Fig. 9:**
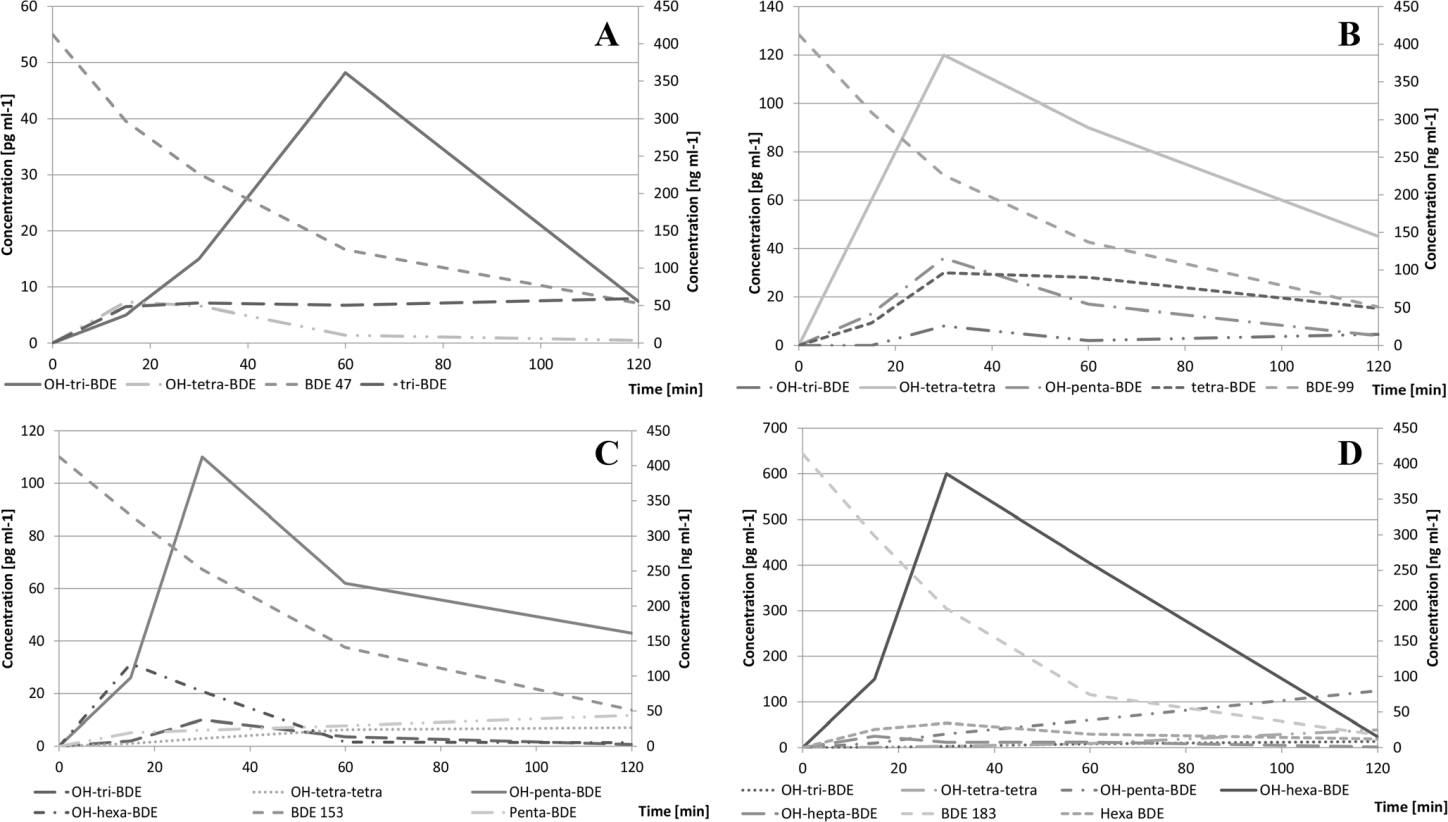
Dependence of concentrations of the identified OH-BDEs (*left axis*), native BDE precursors and debromination products (*right axis*) during UV irradiation of BDE 47 (**a**), BDE 99 (**b**), BDE 153 (**c**), and BDE 183 (**d**)

Hydroxylated BDEs were identified in irradiated BDE aqueous solutions. Time evolution of OH-BDE concentrations formed from tetra- through hepta-brominated BDEs is shown in Fig. 9.

**Fig. 10:**
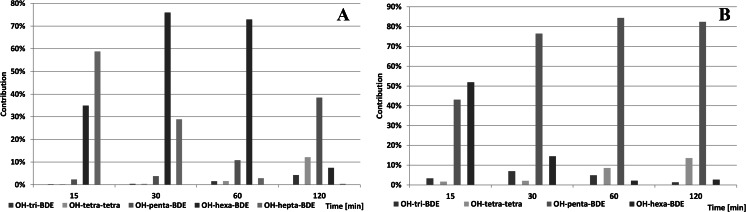
Mean observed profiles of OH-BDEs formed from BDE 183 (**a**) and BDE 153 (**b**)

Profiles of hydroxylated congeners formed from irradiated BDEs 153 and 183 are shown in Fig. 10.

**Fig. 11:**
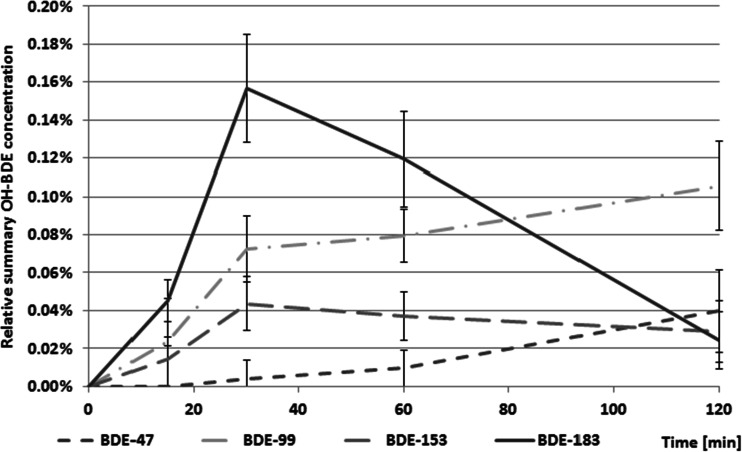
Total concentration of identified OH-BDEs relative to their native BDE precursors

Intensity of OH-BDEs formation in aqueous media was low. Total OH-BDE concentration did not exceeded 0.2 % of the initial precursor mass, see Fig. 11. Presence of lower-brominated congeners indicates subsequent debromination of OH-BDEs or hydro-debromination of degraded congeners. For all tested congeners, the BDE-Br + OH precursor molecule dominated, which suggests that its formation follows the mechanism proposed by Rayne et al. (2006). Hydroxylated compounds with the same degree of bromination observed in the reaction medium suggest that some other reaction ran simultaneously (e.g., synthesis of OH-BDEs from bromophenols formed in the reaction medium).

**Fig. 12:**
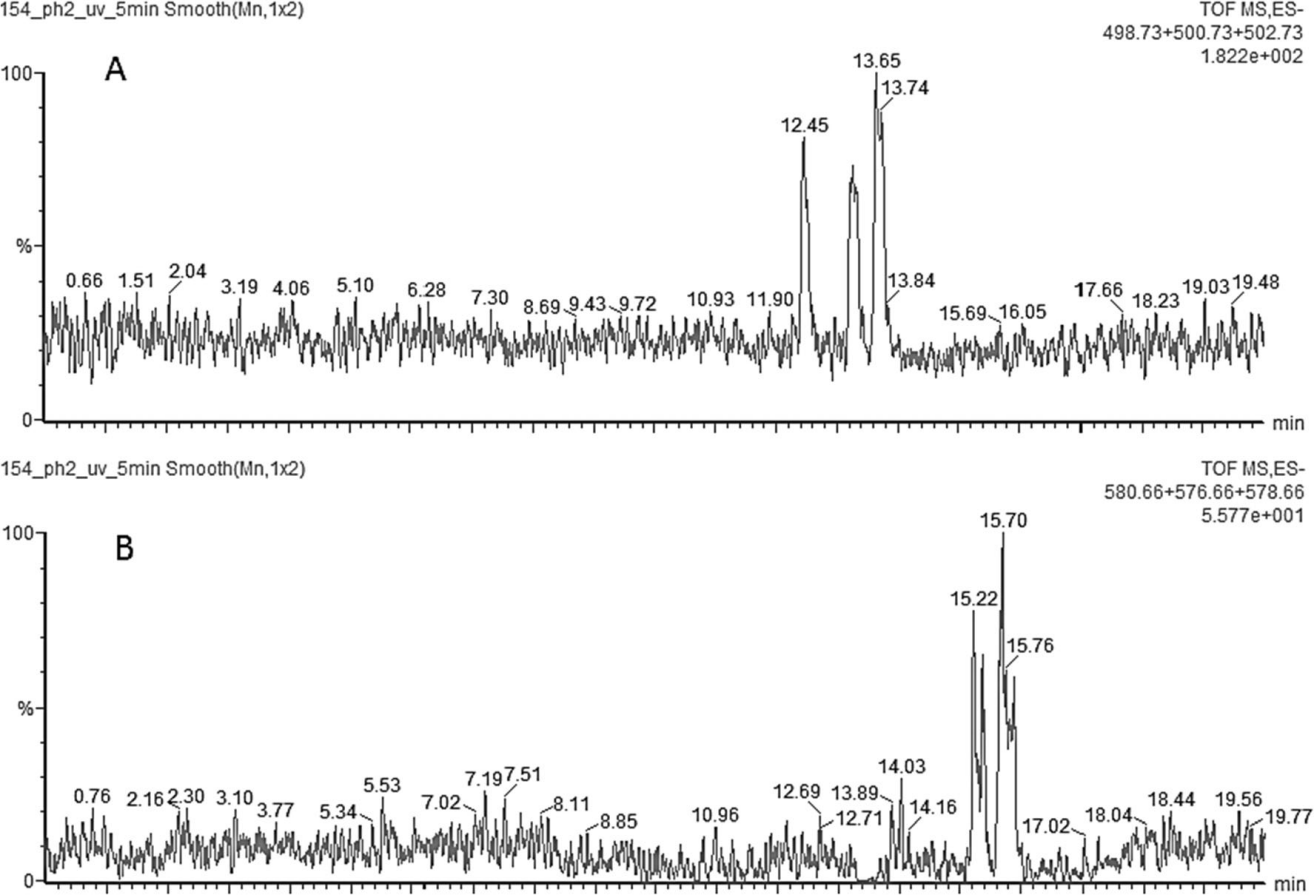
Typical chromatogram of hydroxylated BDEs after 5-min exposure of BDE 154 to UV irradiation. **a** Tetra-brominated BDEs. **b** Penta-brominated BDEs

Concentrations of hydroxylated compounds in the reaction mixtures were generally very low, and they contributed very little to total mass balance of BDE degradation products. They were as unstable under UV irradiation as their native congeners were. The concentrations were affected by pH of the solution: faster degradation rate and lower stability was observed in alkaline medium (pH 10), while in pH 2 medium they were 1.2–3.2 times higher. Most probably, it is a result of a significantly better solubility of OH-BDEs in alkaline media. Those observations are in agreement with Erickson et al. (2012) who pointed out that the number of bromine atoms on the hydroxylated ring also significantly affects dissociation rate, solubility, and ultimately stability of the molecules in the reaction medium. Typical chromatogram of tetra-/penta-brominated OH-BDEs formed under UV irradiation of BDE 154 is shown in Fig. 12.

According to Arnoldsson et al. (2012) and Erickson et al. (2012), ortho-substituted BDEs might act as precursors for brominated dioxins and furans in the UV-catalyzed ring closure reactions. It is unlikely that the latter are mainly formed from anthropogenic BDEs through some (bio) transformations within the environment. Some radiocarbon studies have already proved that those might be of natural origin (Teuten et al. 2005). Observed in this study, relatively low concentrations of OH-BDEs formed from native congeners indicate that the process could not significantly influence biota-observed concentrations. No detectable quantities of brominated dibenzo-p-dioxins nor dibenzo-furans were found in the irradiated samples spiked with native BDEs.

**Fig. 13:**
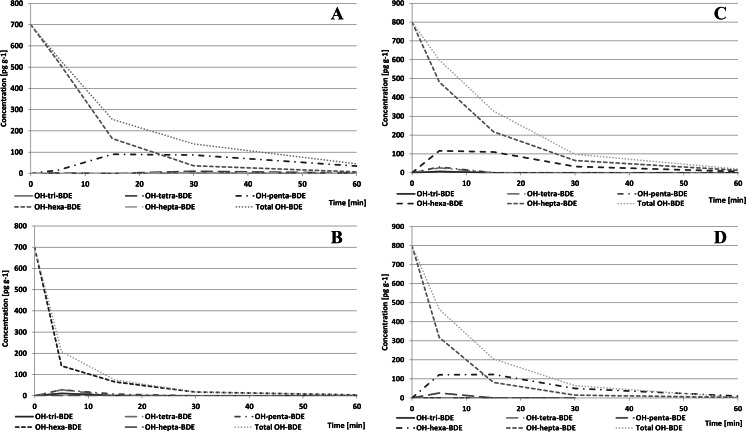
Time dependency of concentration of 6-OH-BDE 137 (**a**–**b**) and 6-OH-BDE 180 (**c**–**d**) and their debromination products under UV irradiation in aqueous solutions at pH 2 (**a**–**c**) and pH 10 (**b**–**d**)

In order to better understand the behavior of OH-BDEs in irradiated water medium, some further studies using tri- to hepta-substituted standards of those compounds were performed. Irradiated OH-BDEs tend to degrade quickly just like native compounds. Half-life times for the studied congeners were in the 6.95–18.62/9.27–33.4-min range for pH 10/2, respectively. The highest half-life times were observed for lower-brominated congeners. The times were lower than for corresponding native congeners. It seems to confirm that OH-BDEs are less stable in alkaline than in acidic solutions (most probably because of better solubility). Erickson et al. (2012) pointed out also higher yield of UV absorption by phenolates. Some de-brominated congeners were identified in the reaction mixtures; however, their concentrations were relatively low, so the bias in OH-BDE concentrations was also low. No significant differences in stability of ortho- and non-ortho OH-substituted BDEs with the same bromination level were observed. Those findings are in agreement with the report of Erickson et al. (2012). Typical relation between irradiation time and concentration of the studied OH-BDEs is shown in Fig. 13. A statistically significant correlation was observed between half-life times and the number of bromine atoms on the OH-BDE molecules (just like in case of native congeners).

Brominated dioxins and at low levels of furans were observed. Most probably, they were formed through elimination of molecular hydrogen bromine (Sanchez-Prado et al. 2012). However, Erickson et al. (2012) have pointed out that such re-arrangement is possible only when the OH-group occupied ortho-position and the opposite ring ortho-position is not occupied. Christiansson et al. (2009) have indicated that such compounds show also relatively high UV absorption rates, which certainly impact their photochemical behaviors. No brominated dioxins nor hydroxylated BDEs were observed in the reaction mixtures containing BDE 209. Watanabe and Tutsukawa (1987) have suggested that brominated furans are formed from some intermediate products of BDE 209 degradation.

**Fig. 14:**
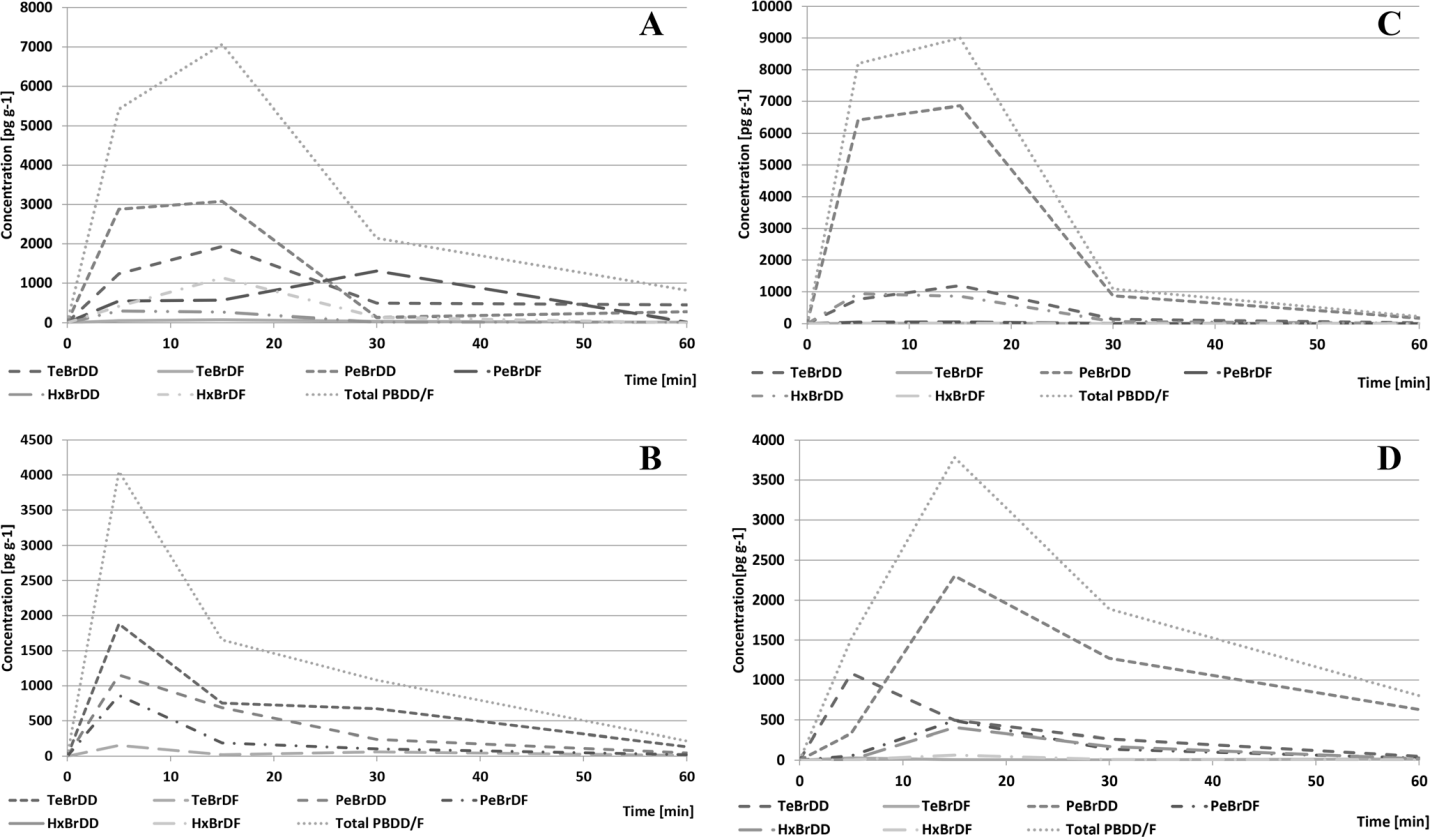
Time dependency of total concentration of the identified PBDD/Fs during UV irradiation of 6-OH-BDE 137 (**a**–**b**) and 6-OH-BDE 180 (**c**–**d**) in aqueous solution at pH 2 (**a**–**c**) and pH 10 (**b**–**d**)

**Fig. 15:**
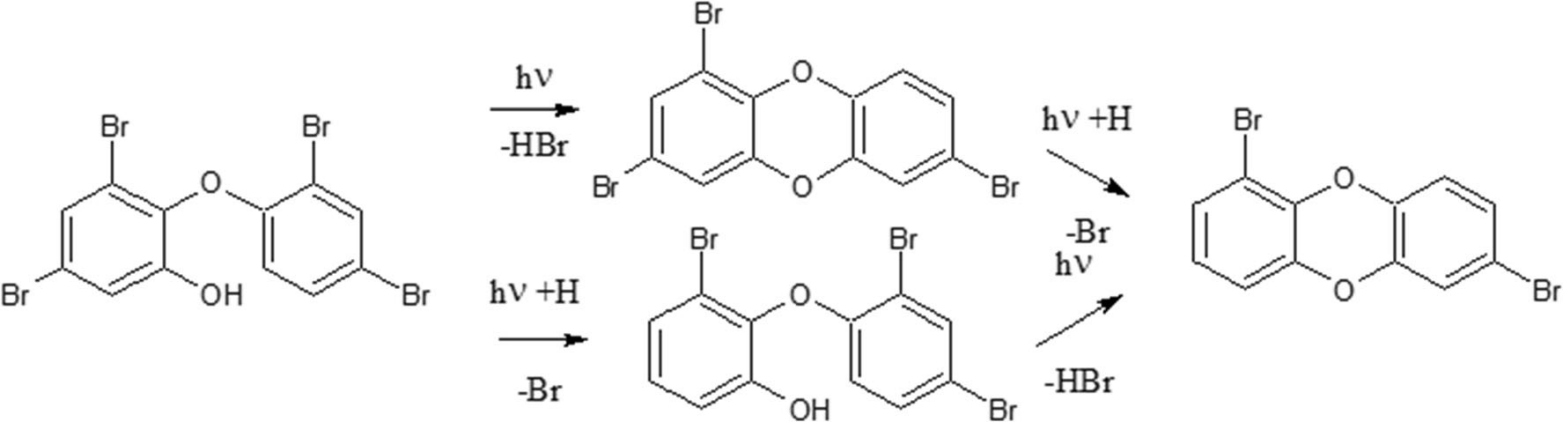
Proposed scheme of PBDDs formation from ortho-substituted OH-BDEs

Only traces of brominated dioxins have been identified in samples containing OH-BDEs not substituted in ortho-position, most probably because of different re-arrangement reactions rather than the proposed cyclisation mechanism. Time dependency of concentration of brominated dioxins in samples containing 6-OH-HxBDE 137 and 6-OH-HpBDE 180 are shown in Fig. 14. Relatively wide spectrum of reaction products indicates that the debrominated OH-BDEs also undergo the cyclization reaction or the formed brominated dioxins are debrominated (Fig. 15).

**Fig. 16:**
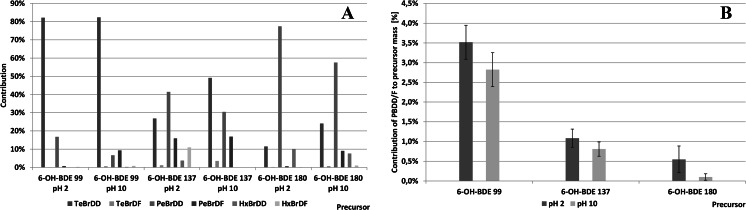
**a** Profiles of PBDD/Fs produced during UV irradiation of various OH-BDE congeners. b PBDD/F mass relative to OH-BDE precursor mass

Dioxin profiles observed in the tested samples are shown in Fig. 16. Lower dioxin concentrations were observed for OH-BDEs in alkaline medium. This observation was in agreement with the report of Erickson et al. (2012). However, the observed brominated dioxins were relatively unstable contrary to the latter authors who suggested that dioxins were much more stable than corresponding OH-BDEs. They reported a broad range of dioxin concentrations relative to precursor mass (0.5–7 %, more than 10-fold differences), which might indicate significantly different stability or synthesis rate of individual congeners. They also found some brominated furans in the reaction mixture, which could indicate that those latter compounds may only be produced via intermediate products of the photodegradation.

Maximum total share of dioxin mass did not exceed 3.5 % of initial precursor mass (Fig. 16), so it seems that the reactions of dioxin synthesis from corresponding OH-BDEs run with a relatively low yield. Assuming a 0.2 % yield of OH-BDE synthesis and 3.5 % yield of OH-BDE-to-dioxins transformation, one can roughly estimate that irradiation of 500 ng of BDE may produce not more than 35 pg of brominated dioxins. Such estimation assumes purely homolytic hydro-debromination reaction leading to formation of a single OH-BDE congener; such assumption has been proven not to be true. Moreover, the ortho-position may not at all be preferentially selected during the hydro-debromination process. The above figures clearly indicate that potential levels of brominated dioxins produced from BDEs at levels found in environmental/biota samples are low.

It must be pointed out that environmentally abundant ortho-substituted OH-BDEs may also originate from sources other than UV irradiation of BDEs. Therefore, levels of brominated dioxins (of which OH-BDEs are potential precursors) in the environment might be much higher than those estimated above. Indeed, higher concentration levels of tri- to tetra-brominated dioxins have been found in low trophy biota (Malmvarn et al. 2005). Levels of brominated dibenzo-furans reported by the latter authors were relatively low. On the other hand, Rose et al. (2015) and Choi et al. (2003) reported only low levels of brominated furans in fish and marine sediments. The available reports are sparse and often contradicting, so no definitive conclusions on transformations of those compounds in the environment may be drawn. Low levels observed in sediments and in fish might indicate lower stability or different distribution paths in biota/environment in comparison to chlorinated analogues. However, that seems unlikely due to structural similarities of both groups of compounds. Further studies are needed to explain the observed differences. Chao et al. (2014) found detectable quantities of brominated furans in marine atmosphere, but no dioxins. That might indicate that elevated levels of brominated dioxins/furans in fish may result from other sources than cyclization of OH-BDEs, for example long-range aerial transport followed by precipitation in water. Significant emissions from incinerated plastics containing PBDEs have been already observed.

## Conclusions

UV irradiation rapidly degraded PBDEs in majority of the investigated cases, mainly by way of debromination. However, debromination was not the main degradation pathway of BDE 209, often observed in the environmental background. BDE 209 seems not to be responsible for lower-brominated BDEs in the environment. Reaction medium strongly affected profiles resulting from debromination.

Reactions performed in organic solvents do not reflect processes occurring in the environment. Hydro-debromination reactions of native BDEs in aqueous media leading to formation of OH-BDEs have been confirmed. However, levels of BDEs observed in the environment and reaction yields are too low to accept those reactions as any significant source of the compounds. OH-BDEs were also found to have low photolytic stability. Reaction yield was affected by pH of the reaction medium.

Under the influence of UV irradiation, ortho-substituted OH-BDEs may form a wide spectrum of brominated dibenzo-p-dioxins and brominated dibenzo-furans. The latter probably result from secondary reactions of bromophenols formed in the first place in the reaction medium.
